# Peroxisome proliferator-activated receptors-mediated diabetic wound healing regulates endothelial cells’ mitochondrial function via sonic hedgehog signaling

**DOI:** 10.1093/burnst/tkaf063

**Published:** 2025-09-10

**Authors:** Shunli Rui, Fugang Xiao, Qin Li, Mengling Yang, Linrui Dai, Shiyan Yu, Xiaoshi Zhang, Xiaoyan Jiang, Seungkuk Ahn, Wenxin Wang, David G Armstrong, Hongyan Wang, Guangbin Huang, Wuquan Deng

**Affiliations:** Department of Endocrinology and Metabolism, Chongqing Emergency Medical Centre, Chongqing University Central Hospital, School of Medicine, Chongqing University, No. 1 Jiankang Road, Yuzhong District, Chongqing 400014, China; Department of Endocrinology and Metabolism, Chongqing Emergency Medical Centre, Chongqing University Central Hospital, School of Medicine, Chongqing University, No. 1 Jiankang Road, Yuzhong District, Chongqing 400014, China; Department of Endocrinology and Metabolism, Chongqing Emergency Medical Centre, Chongqing University Central Hospital, School of Medicine, Chongqing University, No. 1 Jiankang Road, Yuzhong District, Chongqing 400014, China; Department of Endocrinology and Metabolism, Chongqing Emergency Medical Centre, Chongqing University Central Hospital, School of Medicine, Chongqing University, No. 1 Jiankang Road, Yuzhong District, Chongqing 400014, China; Department of Endocrinology and Metabolism, Chongqing Emergency Medical Centre, Chongqing University Central Hospital, School of Medicine, Chongqing University, No. 1 Jiankang Road, Yuzhong District, Chongqing 400014, China; Department of Endocrinology and Metabolism, Chongqing Emergency Medical Centre, Chongqing University Central Hospital, School of Medicine, Chongqing University, No. 1 Jiankang Road, Yuzhong District, Chongqing 400014, China; Department of Endocrinology and Metabolism, Chongqing Emergency Medical Centre, Chongqing University Central Hospital, School of Medicine, Chongqing University, No. 1 Jiankang Road, Yuzhong District, Chongqing 400014, China; Department of Endocrinology and Metabolism, Chongqing Emergency Medical Centre, Chongqing University Central Hospital, School of Medicine, Chongqing University, No. 1 Jiankang Road, Yuzhong District, Chongqing 400014, China; UCD Charles Institute of Dermatology, School of Medicine, University College Dublin, Belfield, Dublin 4 - D04V1W8, Ireland; UCD Charles Institute of Dermatology, School of Medicine, University College Dublin, Belfield, Dublin 4 - D04V1W8, Ireland; Department of Surgery, Keck School of Medicine of University of Southern California, 1510 San Pablo St #514, Los Angeles, CA 90033, United States; Department of Endocrinology and Metabolism, Chongqing Emergency Medical Centre, Chongqing University Central Hospital, School of Medicine, Chongqing University, No. 1 Jiankang Road, Yuzhong District, Chongqing 400014, China; Department of Traumatology, Chongqing University Central Hospital, Chongqing Emergency Medical Center, Chongqing University, No. 1 Jiankang Road, Yuzhong District, Chongqing 400014, China; Department of Endocrinology and Metabolism, Chongqing Emergency Medical Centre, Chongqing University Central Hospital, School of Medicine, Chongqing University, No. 1 Jiankang Road, Yuzhong District, Chongqing 400014, China; School of Life Course and Population Health Sciences, King's College London, Addison House, Guy's Campus, London WC2R 2LS, United Kingdom

**Keywords:** Peroxisome proliferator-activated receptors, Sonic hedgehog, Endothelial function, Oxidative phosphorylation, Diabetic wound healing

## Abstract

**Background:**

Diabetic foot ulcer (DFU) is a common and debilitating complication of diabetes, often leading to delayed wound healing. The peroxisome proliferator-activated receptors (PPARs) play a crucial role in regulating cellular metabolism and promoting angiogenesis. This study aims to elucidate the mechanisms through which the activation of PPARs enhances wound healing, particularly under diabetic conditions, as these mechanisms remain inadequately understood.

**Methods:**

Differentially expressed genes in DFU wounds and normal skin tissues were identified using the GEO database. PPAR expression in DFU neovascularization was validated by quantitative reverse transcription polymerase chain reaction, immunofluorescence, and western blotting. *In vivo*, diabetic mice treated with PPAR agonists (chiglitazar) underwent wound healing assessment, including collagen deposition and angiogenesis. *In vitro*, advanced glycation end-products (AGEs)—induced endothelial cell models were used to evaluate PPAR activation effects on cell migration, tube formation, and mitochondrial function. Whole transcriptome sequencing and mitochondrial analysis were performed to explore the underlying mechanisms, particularly the sonic hedgehog (SHH)–mitochondrial axis.

**Results:**

PPAR expression was significantly downregulated in DFU tissues (*p* < 0.05), and PPAR activation in diabetic mice enhanced wound healing, collagen deposition, granulation tissue proliferation, and angiogenesis (*p* < 0.05). *In vitro*, PPAR activation protected endothelial cells, promoting vascular endothelial growth factor-A (VEGF-A) and CD31 expression, reducing apoptosis, and enhancing cell migration and tube formation (*p* < 0.05). Mechanistically, PPARs activated mitochondrial oxidative phosphorylation and membrane function through the SHH signaling pathway. SHH gene silencing reversed the effects of PPAR activation on mitochondrial function and angiogenesis.

**Conclusions:**

PPAR signaling plays a critical role in DFU healing, with its inhibition linked to vascular dysfunction. Activation of the PPARs/SHH–mitochondrial axis significantly enhances endothelial cell metabolism and angiogenesis. This study provides insights into the molecular mechanisms of diabetic wound healing and supports the clinical potential of PPAR agonists for DFU treatment.

## Graphical Abstract

**Graphical Abstract:**
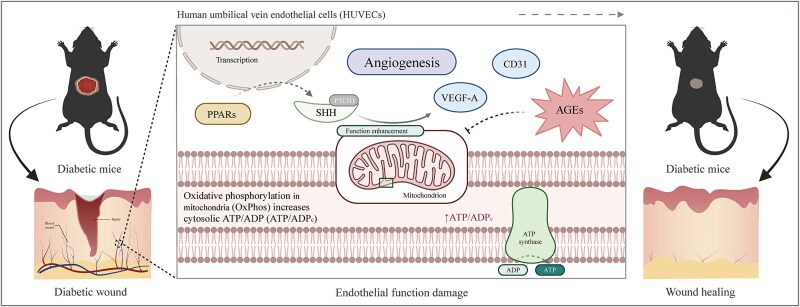

Highlights

- PPARs, as key regulators in the onset and progression of type 2 diabetes, are significantly downregulated in DFU, indicating a long-overlooked therapeutic target. While PPARs play a central role in systemic glucose and lipid metabolism, their involvement in DFU pathophysiology has been largely underexplored.
- Activation of PPARs restores endothelial mitochondrial OXPHOS via the SHH signaling pathway, thereby promoting angiogenesis and tissue regeneration. This establishes a “metabolic bridge” linking systemic metabolic dysfunction to local wound healing.
- Targeting PPARs in DFU not only sheds light on the impaired wound healing observed under hyperglycemic conditions but also redefines metabolic therapeutic strategies. By addressing the pathogenesis of type 2 diabetes at its metabolic core, this study highlights a novel local vascular-metabolic role of PPAR signaling and provides a mechanistic basis for improving wound healing through better metabolic control.

## Background

Diabetic foot ulcer (DFU) constitutes a prevalent and serious complication in individuals with diabetes. The 5-year mortality rate for patients with DFU is ~30%, escalating to >70% for those undergoing major amputation [1, 2]. The rising global prevalence of metabolic disorders is expected to further exacerbate this devastating trend [3]. While effective blood glucose management is a cornerstone strategy for mitigating diabetic complications [4, 5], specific clinical guidelines for recommending hypoglycemic agents to treat DFU itself remain unestablished. Consequently, investigating the ancillary therapeutic benefits of existing glucose-lowering drugs is of paramount importance for broadening their clinical utility and addressing this critical unmet need.

Insulin resistance, implicated as a primary pathological factor in type 2 diabetes, emerges from both diminished glucose uptake in peripheral tissues and unrestrained hepatic glucose production [6]. Counteracting insulin resistance is therefore a core treatment strategy [7]. Currently, peroxisome proliferator-activated receptor (PPAR) agonists are widely deployed as frontline agents for glycemic regulation due to their substantial insulin-sensitizing properties, which effectively decelerate diabetes progression [8]. Beyond their metabolic benefits, PPARs are pivotal nuclear transcription factors that play essential roles in regulating lipid metabolism, adipogenesis, and inflammation [9]. The PPAR family comprises three subtypes—PPARα, PPARβ/δ, and PPARγ—which, upon activation by ligands such as fatty acids or synthetic agonists, form heterodimers with the retinoid X receptor (RXR). This complex translocates to the nucleus, binds to peroxisome proliferator-responsive elements (PPREs), and regulates the expression of genes involved in diverse metabolic processes [10, 11]. Notably, this regulatory cascade extends to mitochondrial biogenesis and function, involving key factors like peroxisome proliferator-activated receptor γ coactivator 1α (PGC-1α), mitochondrial transcription factor A (TFAM), and nuclear respiratory factor 1/2 (NRF1/2), which are crucial for cellular energy homeostasis [12, 13]. Additionally, the heterodimer modulates the expression of genes involved in fatty acid metabolism, such as carnitine palmitoyl transferase 1 (CPT1) and acyl-CoA oxidase 1 (ACOX1), indirectly influencing mitochondrial fatty acid utilization and energy production [14, 15]. Despite a well-defined role for PPARs in systemic metabolism, their potential function in local wound healing processes, particularly in the context of DFU, is poorly understood and constitutes a significant knowledge gap. Impaired angiogenesis and endothelial dysfunction are hallmarks of the non-healing diabetic wound. While PPAR activation is known to influence vascular health and inflammatory responses [11, 16], its specific targets and mechanisms in the diabetic wound microenvironment, especially concerning the regulation of endothelial cell mitochondrial function, remain inadequately elucidated.

In this work, we hypothesized that PPAR activation promotes diabetic wound healing by directly enhancing endothelial cell function via a novel molecular pathway. We found that PPAR expression is significantly downregulated in DFU tissues and that their activation with the pan-agonist chiglitazar dramatically accelerates wound closure in diabetic mice by enhancing angiogenesis, collagen deposition, and epithelialization. Our mechanistic investigations reveal that this effect is mediated through the sonic hedgehog (SHH) signaling pathway, which acts as a critical downstream effector of PPARs to restore mitochondrial oxidative phosphorylation (OXPHOS) in endothelial cells. This study unveils the PPARs/SHH–mitochondrial axis as a novel regulatory mechanism critical for diabetic wound healing, providing a strong rationale for repurposing PPAR agonists as a promising therapeutic strategy for DFU.

## Methods

### Gene Expression Omnibus

The GEO database (https://www.ncbi.nlm.nih.gov/geo/) was utilized to conduct a differential expression analysis of the GSE134431 dataset [17]. This analysis involved a comparative work between skin wounds from nondiabetic patients and skin tissues from patients with DFU, aiming to elucidate the molecular mechanisms and transcriptional networks underlying dysregulation in DFU. Data analysis and visualization were carried out using SangerBox (http://sangerbox.com).

### Collecting samples of human tissue

Diabetic and nondiabetic foot surgery patients’ skin, including four normal skin tissue samples and four DFU skin tissue samples, was studied. In the following steps, snap frozen and stored at −80°C, the tissues were subjected to quantitative reverse transcription polymerase chain reaction (qRT-PCR) analysis or embedded in paraffin for further examination. The study protocol was reviewed and approved by the Ethics Committee of the Chongqing Emergency Medical Center, and it was all conducted in accordance with the Declaration of Helsinki’s ethical standards.

### Chemical preparation

Chiglitazar (Chi) was discovered and synthesized by Chipscreen Biosciences Ltd (Beijing, China). Pemafibrate, rosiglitazone (Ros), and GW501516 were purchased from Target Molecule Corporation (Boston, MA). All chemicals were supplied with purity qualified.

### Cell culture and cell viability

Human umbilical vein endothelial cells (HUVECs) were procured from Procell Company (Wuhan, China). The 1640 medium, under conditions of 37°C in a humidified incubator with 5% CO_2_, was used to maintain the culture of the cells (12633012; Gibco). The culture medium was supplemented with 1% penicillin and streptomycin (BaiDi Biotechnology Co., Ltd (BDBIO)) and 10% fetal bovine serum (JYK-FBS-301; Inner Mongolia Jin Yuan Kang Biotechnology Co., Ltd). All experimental procedures were performed using HUVECs at passages 3–6.

Cell viability was assessed using the Cell Counting Kit-8 (CCK-8, CP0001; CYTOCH) following its protocol, with readings taken on a Synergy™ H1 microplate reader.

### Tube formation

HUVECs were assessed using matrix gels (356234; Corning) to assess their angiogenic potential. During the incubation period, 70 L of liquid gel was evenly distributed among the 48 wells while being kept on ice. Subsequently, to solidify the gel, the plates were heated to 37°C for 30 min. In the post-incubation step, pretreated HUVECs were seeded onto the gel that had been solidified. Microscopy was then used to monitor and document tube formation over the course of 6 h.

### HUVEC migration

Cells were cultured in 6-well plates and pretreated with 200 μg/ml advanced glycation end-products (AGEs) for a duration of 24 h [18]. Following this pretreatment, the cells were exposed to 1 μM Chi for an additional 48 h. A scratch assay was performed by introducing a linear wound using a 20-μl pipette tip. Biotek’s Lionheart FX automated microscope (USA) was used to track cellular responses for 24 h.

Cells were pretreated with 200 μg/ml AGEs for 24 h, followed by exposure to 1 μM Chi for an additional 48 h. Subsequently, cells were plated in 100 μl of serum-free medium at a density of 4 × 10^5^ cells per 1 ml into the plates with Transwell chambers (724301; NEST Biotechnology). Medium containing 10% fetal bovine serum was filled into the lower compartments. A 24-h incubation period was followed by the removal of the Transwell plates, and paraformaldehyde 4% was applied to the cells for 15 min to fix them. We stained the chambers with Coomassie Brilliant Blue for 15 min, rinsed them with PBS, removed debris with cotton swabs, and dried them at room temperature. Under an inverted microscope, these samples could then be visualized, photographed, and counted.

### qRT-PCR analysis

Total RNA was isolated employing the TRIzol™ reagent (Invitrogen). Complementary DNA (cDNA) synthesis was conducted utilizing the High-Capacity cDNA Reverse Transcription Kit (Invitrogen). Subsequent qRT-PCR was performed with the 2X Universal SYBR Green Fast qPCR Mix (RK22203; ABclonal, China), and data analysis was executed using the CFX™ 96 Manager Software (Bio-Rad). The expression levels of mRNA were normalized to β-actin and quantified relative to this reference gene. Primer sequences are described in Table S1.

### Flow cytometry

As recommended by the manufacturer, the Apoptosis Analysis Kit (FXP023; 4Abio) was used to analyze apoptosis in our cells. MitoTracker™ Red CMXRos (M7513; Thermo Fisher Scientific), JC-10 for mitochondrial membrane potential (FXP134; 4Abio), mitochondrial permeability transition pore (mPTP) staining (KTA4002; Abbkine), and mitochondrial ATP fluorescent probe (D2606; Beyotime) were also conducted following the provided protocols.

### TUNEL assay

In comparisons between the groups, TUNEL Apoptosis (KGA702; KeyGen Biotech) was used in order to determine apoptosis levels, in accordance with specified protocol.

### Transcriptome sequencing

Cells or tissues were harvested using RNAiso Plus (9109; Takara) to extract total RNA as per the specified protocol. Eukaryotic mRNA was enriched with Oligo (dT) magnetic beads. After RNA fragmentation, cDNA libraries were created and the Illumina NovaSeq 6000 was used for sequencing RNA. In FPKM, gene expression was measured. Differential expression was deemed significant with an adjusted *P*-value <0.05 and a log_2_ fold change ≥1.3.

### Knockdown of SHH in HUVECs

Transfections with siRNA were carried out using EndoFectin™ RNAi (EF021; GeneCopoeia). The nucleotide sequence of the SHH siRNA utilized in this work was 5′-CCAGAAACTCCGAGCGATT-3′.

### Western blot analysis

Proteins were extracted from cells using an inhibitory RIPA buffer (SC-364162; Santa Cruz), and a phosphatase inhibitor (B15001; Selleck) was used to generate the sample and to determine the results. For the extraction of mitochondrial membrane proteins, the procedure outlined in the cell mitochondria isolation kit (C3601; Beyotime) was followed. A BCA protein detection assay kit (ZJ102; Epizyme) was used to determine the protein concentration. An electrophoretic polyacrylamide gel was used to electrophorese proteins, which were then transferred onto PVDF membranes (Millipore, Billerica) with protein marker (20350; Yeasen). The membrane was blocked for 1 h with a TBST solution containing 5% milk after incubated overnight at 4°C with primary antibodies against PPARα (340843; Zen Bio), PPARγ (AF7797; Beyotime), PPARδ (AF7800; Beyotime), CD31 (R10021; Zen Bio), PTCH1 (C53A3; CST), GLI1 (C68H3; CST), SHH (C9C5; CST), VEGF-A (WL00009b; WanLeiBio), Bax (AG1208; Beyotime), Bcl-2 (AF6139; Affinity Bioscience), Cleaved caspase-3 (340843; Zen Bio), OXPHOS (PK30006; Proteintech), and β-tubulin (10094-1-AP; Proteintech). After washing with TBST about three times, the secondary antibodies were incubated with horseradish peroxidase (1430; Thermo Fisher Scientific). Antigen detection was subsequently performed using a chemiluminescence (ECL) kit (SB-WB004; Share-bio, Shang Hai). Immunoreactive bands were visualized using the ChemiDoc Touch imaging system (Bio-Rad, USA) and analyzed with ImageJ software.

### Immunofluorescent and morphological staining

Cells cultured on coverslips were fixed using 4% paraformaldehyde in PBS for 10 min. After post-fixation, the cells were washed with PBS and then permeabilized in PBG–Triton solution (consisting of 0.5% Triton, PBS, 0.5% BSA, and 0.4% fish skin gelatin) for 1 h. Following permeabilization, the cells were incubated overnight at 4°C with the primary antibody. After washing with PBS, the cells were incubated at room temperature for 1 h with the secondary antibody. Finally, as formulated by Thermo Fisher Scientific, the coverslips were mounted with an antifade mountant containing DAPI. Following fixation of the tissues with paraformaldehyde for 16 to 24 h at 4°C, 5-μm sections were prepared. These sections underwent deparaffinization and antigen retrieval using sodium citrate, and a paraffin embedding step is followed to continue the processing of the specimen. A blocking buffer was then applied to the sections. At 4°C overnight incubated with antibodies specific to PPARα (340843; Zen Bio), PPARγ (AF7797; Beyotime), PPARδ (AF7800; Beyotime), CD31 (R10021; Zen Bio), α-SMA (db12547; Diagbio), SHH (CSB-PA623000LA01HU; Huamei Biotech), and VEGF-A (WL00009b; WanLeiBio). It was performed using secondary antibodies conjugated to Alexa Fluor 488 and 555 (Thermo Fisher). DAPI was used as a staining agent in the mounting medium (SI103; Seven/Abcells). In accordance with the manufacturer’s instructions, Masson trichrome staining was performed using a kit (G1346; Solarbio). Collagen was quantified in stained skin sections using ImageJ software.

### Mitochondrial oxygen consumption rate assay

HUVECs were plated at a density of 1.25 × 10^4^ cells/well in specialized XF96 microplates (Agilent Technologies, USA) and maintained under standard culture conditions (37°C, 5% CO_2_) for 12–16 h. Sensor probes underwent preparatory hydration 12 h prior to experimentation using manufacturer-supplied calibration solution in 37°C atmospheric environment devoid of carbon dioxide. During experimental execution, cellular monolayers underwent triple-rinse purification followed by immersion in XF base medium supplemented with appropriate metabolic substrates. Then, they underwent temperature equilibration (37°C, non-CO_2_ environment) for 60 min preceding analysis. Using the XF96 extracellular flux analyzer, sequential administration of metabolic modulators (oligomycin, FCCP, and rotenone/antimycin A) was implemented.

### RNA fluorescence *in situ* hybridization

The fluorescence *in situ* hybridization (FISH) assay was conducted to investigate the expression of SHH and CD31 within tissue sections, utilizing the FISH kit (Servicebio, Wuhan, China) alongside specific detection probes for SHH and CD31 (Servicebio). Initially, the tissue sections underwent dewaxing, rehydration, and repair processes, followed by protease K digestion for 25 min at 37°C. Pre-hybridization was performed with a hybridization solution at 40°C for 1 h. Subsequently, the tissue sections were incubated overnight at 40°C with either the SHH probe or the CD31 probe to facilitate hybridization. Post-hybridization, the sections were washed with SSC washing buffer at 40°C, and fluorescence signals were enhanced using a Cy3-conjugated label probe for CD31 detection or an Alexa Fluor 488–conjugated label probe for SHH. Finally, the sections were counterstained with DAPI. Fluorescent images of the tissue sections were captured using confocal fluorescence microscopy (Olympus, Japan), and mean fluorescence intensities were quantified using ImageJ software (version 1.8.0). The sequences of the probes are provided in Table S1.

### T2DM induction

Mouse male C57BL/6Js were provided by Seye Biotech (Suzhou, China), which were kept on a 12-h light/dark cycle with free access to standard chow and water. The experimental design employed eight animals per treatment group. The Chongqing University Research Animal Care Committee approved the study protocol (COU-IACUC-RE-202308-004). For 4 weeks on a high-fat diet (D12492; Research Diets), the mice received daily intraperitoneal injections of 35 mg/kg streptozotocin (STZ) dissolved in citrate buffer (pH 4.2–4.5) for 5 days. Blood samples from the tail were used to measure glucose levels. In mice with blood glucose levels above 16.7 mmol/L after a week, the animals were categorized as diabetic [19, 20].

### Skin wound models

According to the method described, an experimental wound healing model was created [21]. Animals were anesthetized with isoflurane (RDW), and a 6-mm cylindrical biopsy tool (Health Link, Florida) was used to make the dorsum both sides with full-thickness wounds. Following surgery, digital images of the wounds were taken every other day. Standardized calibration protocol was utilized for the measurement of wound areas, and the wound closure rate was calculated using ImageJ software (NIH, Bethesda, MD). To understand wound healing mechanisms, mRNA and protein analyses were performed on euthanized mice. Using 8-mm skin biopsy punches, wound tissues were collected.

### Statistics

We used the GraphPad Prism 9.3 software (San Diego, CA) for statistical analysis of the supplementary data. The central tendency of the data is represented by the mean value. The data are presented as mean ± standard deviation (SD), and this is clearly indicated in the corresponding figure legends. The sample size (*n*) for each study is described in the corresponding figure legend. Student’s *t* test was employed for comparisons between two groups, while for multiple group comparisons, analysis of variance followed by *post hoc* Tukey test was applied. Differences were considered significant when denoted as ^*^p < 0.05, ^**^p < 0.01, ^***^p < 0.001.

## Results

### Dysregulation of PPAR expression in DFU

**Figure 1:**
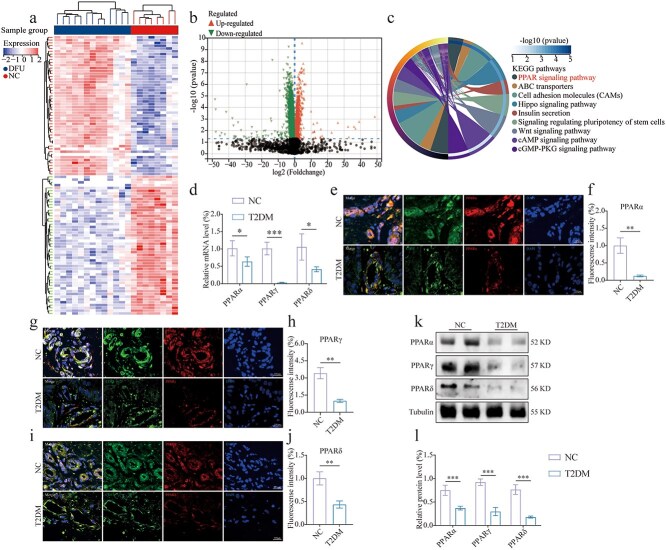
Dysregulation of PPARs expression in DFU. (**a**) Heat map of DEGs (adj. *p* < 0.01 based on sequencing results). (**b**) Volcano map of DEGs (adj. *p* < 0.01 based on sequencing results). (**c**) KEGG pathway enrichment of DEGs. (**d**) A qRT-PCR analysis was conducted to evaluate the relative mRNA expression levels of PPARs in the skin, comparing healthy individuals to patients diagnosed with T2DM, *n* = 4. (**e, f, g, h, i, j**) Representative immunofluorescence images and analyses of PPARs (labeled with Alexa Fluor 555) expression levels in skin neovascularization (CD31, labeled with Alexa Fluor 488), comparing normal individuals to patients with T2DM. The cell nucleus was stained with DAPI (blue), *n* = 3 (scale bar: 100 μm). (**k, l**) The relative expression levels of PPARs in skin tissue were compared between normal individuals and patients with T2DM using western blot analysis, *n* = 4. The results are expressed as mean ± SD. ^*^p < 0.5, ^**^p < 0.01, ^***^p < 0.01; ns, not significant. *DFU* diabetic foot ulcer, *SD* standard deviation

To identify potential biomarkers and therapeutic targets, we compared skin wounds from nondiabetic patients with skin tissue from patients with DFU. Differential expression analysis of the GSE134431 dataset retrieved from the GEO database identified 225 significantly upregulated and 876 downregulated genes using stringent thresholds (|log2 fold change| > 1.5, adj. *p* < 0.01; Figure 1a, b). Subsequent KEGG pathway enrichment analysis revealed 19 significantly altered signaling pathways (Benjamini–Hochberg corrected *p* < 0.05), with the PPAR signaling pathway demonstrating particularly prominent dysregulation (*p* < 0.01; Figure 1c). Preliminary verification on skin samples from healthy individuals and DFU, conducted via qRT-PCR, confirmed a significant downregulation of the PPAR family in DFU (Figure 1d). Additionally, tissue immunofluorescence analysis demonstrated that PPAR expression was inhibited in skin neovascularization in DFU (Figure 1e–j). Subsequent validation using western blot further corroborated the low expression of PPARs in DFU (Figure 1k, l). These results have suggested that the expression of the PPARs has been significantly inhibited in the formation of neovascularization in DFU, indicating that PPARs may have played a crucial role in diabetic wound healing.

### Activating PPAR signaling enhances wound healing in diabetic mice

**Figure 2:**
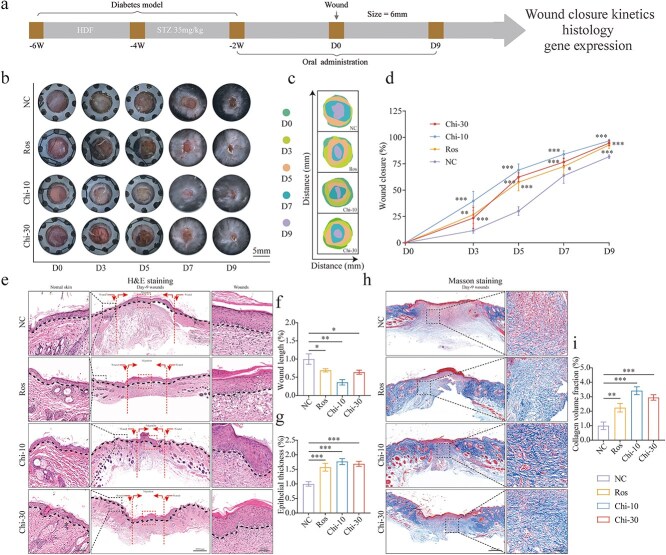
Activating PPAR signaling enhances wound healing in diabetic mice. (**a**) A systematic evaluation protocol for the assessment of skin wound healing. (**b, c**) Images and mode patterns depicting wound closure in the NC, Ros (10 mg/kg), Chi-10 (10 mg/kg), and Chi-30 (30 mg/kg) groups at days 0, 3, 5, 7, and 9 post-operation. (**d**) Wound closure rates were quantified using ImageJ software by calculating the percentage of closure relative to the day 0 wound size, and comparisons were made between the Ros, Chi-10, and Chi-30 groups against the NC group, *n* = 10. (**e, f, g**) Quantitative assessment of neo-epithelium gap width and re-epithelization degree, marked by horizontal black lines, *n* = 3 (scale bar: 1000 μm). (**h, i**) Cutaneous wound sections were subjected to Masson’s trichrome staining, *n* = 3 (scale bar: 1000 μm). The results are expressed as mean ± SD. ^*^P < .05, ^**^P < .01, ^***^P < .001; *ns*, not significant. *PPAR* peroxisome proliferator-activated receptor, *Ros* rosiglitazone, *SD* standard deviation

To explore the role of PPAR activation on DFU, we used C57BL/6J mice. We summarized the workflow course (Figure 2a), which assessed the healing of diabetic wounds. During the model development phase, we documented the blood sugar changes before and after the STZ intervention and the weight changes of the high-fat diet (Figure S1a, b, see online supplementary material). Concurrently, the reliability of the diabetic mouse model was confirmed using immunofluorescence analysis to examine pancreatic tissue injury 7 days post-STZ intervention (Figure S1c, d, see online supplementary material). In this work, further experiments were conducted on mice exhibiting persistently high blood sugar levels consistent with diabetes standards. To evaluate wound healing, we aimed to minimize contraction in the rodent model by simulating the skin surface healing process and applying a silicone gel pad to achieve an anti-contraction effect. The images and pattern photographs demonstrated healing process following the administration of a single PPARγ activation and the pan-activation of PPARs (Figure 2b, c). Quantitative evaluations of wound healing rates indicated that the activation of PPARs significantly promoted diabetic wound healing compared to the NC group and the group receiving a single subtype activation, with the maximal effect observed at a dose of 10 mg/kg (Figure 2d). Quantitative analysis of wound length (indicated by the horizontal black line in H&E staining images) and the extent of re-epithelialization revealed that PPAR activation was considerably more effective in promoting wound regeneration and re-epithelialization than PPARγ activation alone (Figure 2e–g). The quantification of collagen deposition volume using Masson’s trichrome staining showed that PPAR activation also enhanced collagen production in comparison to both the control group and the single PPARγ group (Figure 2h, i). Collectively, these results have suggested that PPAR activation has significantly improved the healing of diabetic wounds.

### P‌PAR signaling activation enhances angiogenesis *in vivo*

**Figure 3:**
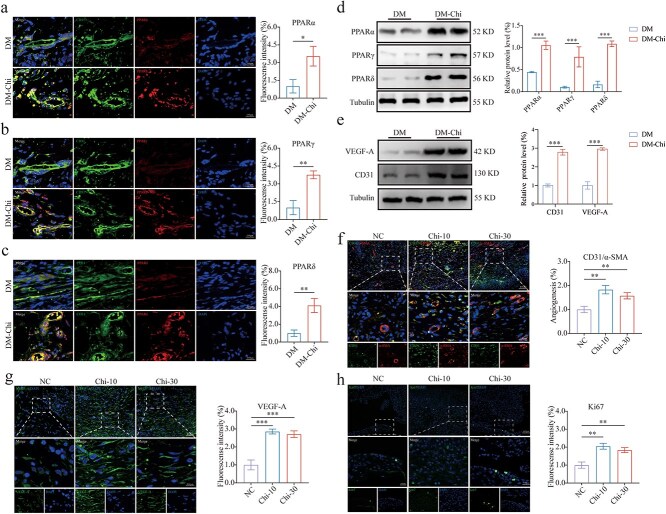
PPAR signaling activation enhances angiogenesis *in vivo*. (**a, b, c**) Representative immunofluorescence images and analyses for detection of PPARs (labeled with Alexa Fluor 555) expression levels in neovascularization (CD31, labeled with Alexa Fluor 488) of skin wounds on day 9 of the diabetic wound healing model after Chi intervention, *n* = 3 (scale bar: 100 μm). (**d**) The relative level of PPAR expression in skin tissue during the diabetic wound healing model after Chi intervention was detected by western blot, *n* = 4. (**e**) The relative level of CD31 and VEGF-A expression in skin tissue during the diabetic wound healing model after Chi intervention was detected by western blot, *n* = 4. (**f**) Images and quantification of immunofluorescence staining for CD31 and α-SMA to reflect the degree of neovascularization, *n* = 3 (scale bar: 400 μm). (**g**) Images and quantification of immunofluorescence staining for VEGF-A in skin wounds on day 9 after Chi intervention, *n* = 3 (scale bar: 400 μm). (**h**) Representative immunofluorescence images and analyses for Ki67 in skin wounds on day 9 after Chi intervention, *n* = 3 (scale bar: 400 μm). The results are expressed as mean ± SD. ^*^p < 0.05, ^**^p < 0.01, ^***^p < 0.001; ns, not significant. *PPAR* peroxisome proliferator-activated receptor, *Ros* rosiglitazone, *SD* standard deviation, *VEGF-A* vascular endothelial growth factor A

Given the role of PPAR activation in promoting diabetic wound healing, we first confirmed increased PPAR expression in neovascularization during diabetic wound healing via immunofluorescence (Figure 3a–c). Subsequent analyses verified an upregulation of PPAR protein expression at the wound site (Figure 3d) alongside an increased expression of RXRα protein (Figure S1e, f, see online supplementary material), determined by western blot analysis. These findings suggest that Chi activates PPARs. Additionally, elevated levels of CD31 and VEGF-A proteins were detected through western blot analysis, as depicted in Figure 3e. To further understand the effects of PPAR activation, we examined granulation apoptosis, proliferation, and angiogenesis during wound healing in diabetic wound edge. Immunofluorescence analysis revealed that PPAR activation significantly reduced apoptosis at the wound edges (Figure S1g, h, see online supplementary material). Angiogenesis was assessed using CD31 and α-SMA co-staining as indicators for endothelial and smooth muscle cells, respectively. Image analysis and statistical evaluation indicated that PPAR activation promoted angiogenesis, highlighting its potential application in blood vessel growth and tissue repair (Figure 3f). Additionally, we observed enhanced VEGF-A secretion following PPAR activation (Figure 3g). Ki67 immunohistochemical staining demonstrated enhanced cellular proliferation, further supporting the therapeutic potential of PPAR activation in promoting diabetic wound healing (Figure 3h). These results have suggested that PPAR activation may have enhanced vascular remodeling and repair, which could be integral to its therapeutic efficacy in improving diabetic wound healing.

### Enhance the functionality of HUVECs by activating PPAR signaling *in vitro*

**Figure 4:**
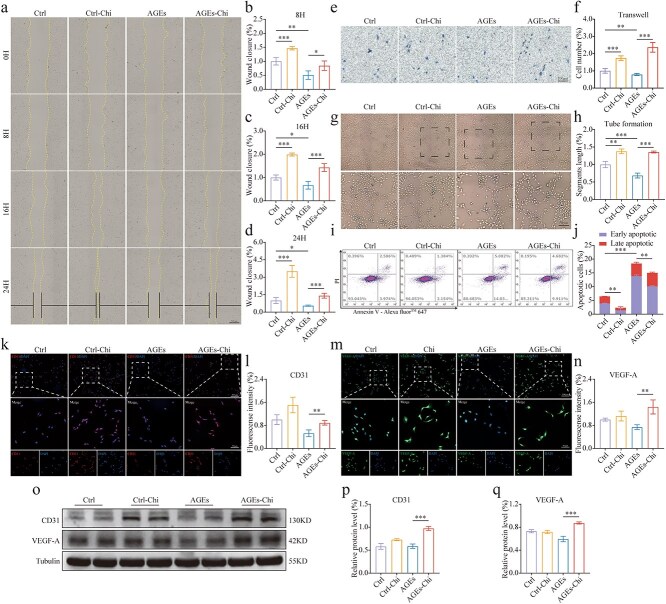
Enhancing the functionality of HUVECs by activating PPARs signaling *in vitro*. Cells were pretreated with 200 μg/ml AGEs for 24 h, followed by exposure to 1 μM Chi for an additional 48 h. (**a, b, c, d**) Cell scratch assays were observed in living cells at 0, 8, 16, and 24 h, *n* = 4 (scale bar: 500 μm). (**e, f**) Transwell assays were conducted for evaluation of HUVEC migration, *n* = 4 (scale bar: 100 μm). (**g, h**) Tube formation assays were conducted to measure segment length and evaluate HUVECs’ tube formation capability, *n* = 3 (scale bar: 100 μm). (**i, j**) HUVEC cell apoptosis were measured by flow cytometry, *n* = 3. (**k, l**) Representative immunofluorescence images and analyses for CD31 in HUVECs after intervention, *n* = 3 (scale bar: 200 μm). (**m, n**) Representative immunofluorescence images and analyses for VEGF-A in HUVECs after intervention, *n* = 3 (scale bar: 200 μm). (o, p, q) The relative levels of CD31 and VEGF-A in HUVECs after intervention were measured by western blot, *n* = 4. The results are expressed as mean ± SD. ^*^p < 0.05, ^**^p < 0.01, ^***^p < 0.001; ns, not significant. *PPAR* peroxisome proliferator-activated receptor, *HUVECs* human umbilical vein endothelial cells, *Ros* rosiglitazone, *AGEs* advanced glycation end-products, *VEGF-A* vascular endothelial growth factor A

Given the significant effect of PPAR activation on angiogenesis *in vivo*, we investigated its influence on HUVECs function *in vitro*. A gradient administration experiment was designed to determine the optimal concentration of PPAR activator in each subtype of HUVECs using western blot analysis. The optimal concentration for activating all PPAR subtypes in cells was found to be 1 μM (Figure S2a, see online supplementary material). To simulate a hyperglycemic environment in endothelial cells, AGEs were administered at a constant concentration of 200 μg/ml. This dose decreased the expression of all PPAR subtypes, but this reduction was reversed upon the administration of PPAR activator, validating the pathological model and the efficacy of the activator (Figure S2b, c, see online supplementary material). To further explore the effects of PPAR activation on angiogenesis, we examined the migration capacity of HUVECs using cell scratch and Transwell assays. The migration process was continuously monitored for 24 h. A black horizontal line indicated the degree of wound healing (Figure 4a). In the scratch assay, we monitored cell migration at 8, 16, and 24 h. Compared to the normal control group, the wound closure rates in the presence of AGEs were reduced by approximately 49%, 34%, and 45%, respectively. Notably, following the intervention of PPAR activation, the impairment was remarkably improved at all observed points under both physiological and pathological conditions (Figure 4b–d). As expected, the Transwell assay confirmed the results of the scratch test (Figure 4e, f). Additionally, tube formation assays showed that PPAR-activated cells formed significantly longer tube segments, particularly under simulated pathological conditions (Figure 4g, h). We also investigated the effect of PPAR activation on apoptosis. Flow cytometry and TUNEL staining confirmed that PPAR activation exhibited a significant antiapoptotic effect (Figure 4i, j; Figure S2d, e, see online supplementary material). We also performed western blot analysis on apoptosis in the pathological state, revealing antiapoptotic changes in Bcl-2, Bax, and Cleaved Caspase-3 following PPAR activation (Figure S2f, g, see online supplementary material). Additionally, cellular immunofluorescence was utilized to quantitatively analyze the expression of key functional factors in HUVECs, including CD31 and VEGF-A levels. The results demonstrated that PPAR activation significantly promoted cell function (Figure 4k–n). Furthermore, western blot analysis corroborated its enhancing effect on endothelial cell function (Figure 4o–q). Overall, these results have suggested that activating PPARs has effectively inhibited AGE-induced functional impairment of HUVECs.

### Activation of PPAR signaling enhances OXPHOS

**Figure 5:**
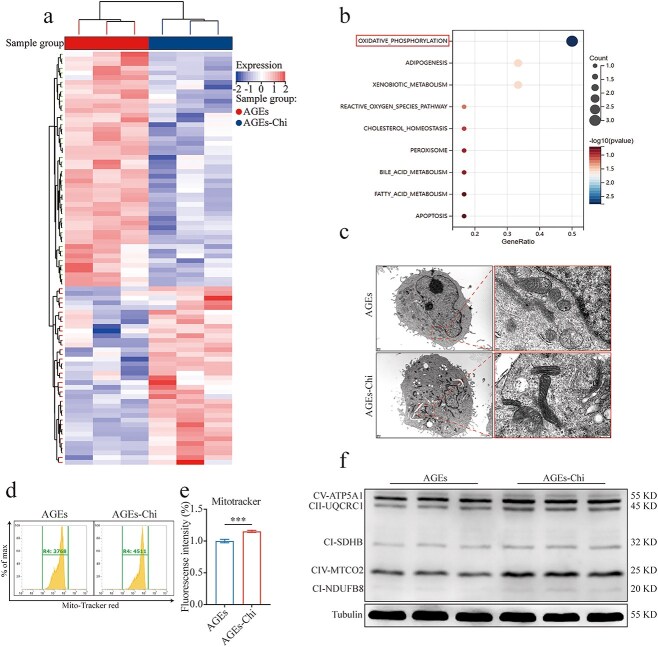
Activation of PPAR signaling enhances OXPHOS. Cells were pretreated with 200 μg/ml AGEs for 24 h, then exposed to 1 μM Chi for 48 h. (**a**) Heat map of DEGs (adj. *P*-value <.01 based on sequencing results). (**b**) KEGG pathway enrichment of mitochondrial DEGs. (**c**) The morphology of mitochondrial was viewed by transmission electron microscope. (**d, e**) MitoTracker™ Red CMXRos was measured by flow cytometry after intervention, *n* = 3. (**f**) The relative levels of mitochondrial OXPHOS in HUVECs after intervention were measured by western blot, *n* = 3. The results are expressed as mean ± SD. ^*^p < 0.05, ^**^p < 0.01, ^***^p < 0.001; ns, not significant. *PPAR* peroxisome proliferator-activated receptor, *HUVECs* human umbilical vein endothelial cells, *OXPHOS* oxidative phosphorylation, *AGEs* advanced glycation end-products, *SD* standard deviation

To elucidate how PPAR activation enhances HUVEC function, we first assessed cell viability in response to PPAR activator under both physiological and pathological conditions, which determined their therapeutic effects under pathological states (Figure S3a, b, see online supplementary material). Given our focus on therapeutic effects within pathological environments, we performed whole transcriptomic sequencing post–cell intervention under these conditions (adj. *p* < 0.01; Figure 5a). KEGG analysis of DGEs highlighted the profound impact of PPAR activation on OXPHOS in endothelial cells (Figure 5b). Previous studies have established the connection between PPAR activation and energy metabolism, including β-oxidation and OXPHOS [22–24]. Consequently, we further investigated the effect of PPAR activation on mitochondrial function in pathological states. Using mitochondrial electron microscopy, we observed beneficial morphological changes in AGE-damaged mitochondria post–PPAR activation, such as reduced vacuolation and increased cristae density (Figure 5c). Additionally, the flow cytometry of MitoTracker™ Red CMXRos assay indicated a significant increase in mitochondrial activity following PPAR activation (Figure 5d, e). Other mitochondrial function indicators corroborated these findings. mPTP assay confirmed the protective effect of PPAR activation on mitochondria (Figure S3c–f, see online supplementary material), and JC-10 assay detection results were consistent with these observations (Figure S3g, h, see online supplementary material). Western blot analysis further demonstrated upregulation of key regulatory proteins involved in mitochondrial OXPHOS post–PPAR activation in pathological conditions, and this tendency was further validated in diabetic wounds (Figure 5f; Figure S3j, see online supplementary material). Collectively, these results have suggested that PPAR activation has significantly enhanced mitochondrial function.

### P‌PAR signaling promotes HUVEC function via SHH expression

**Figure 6:**
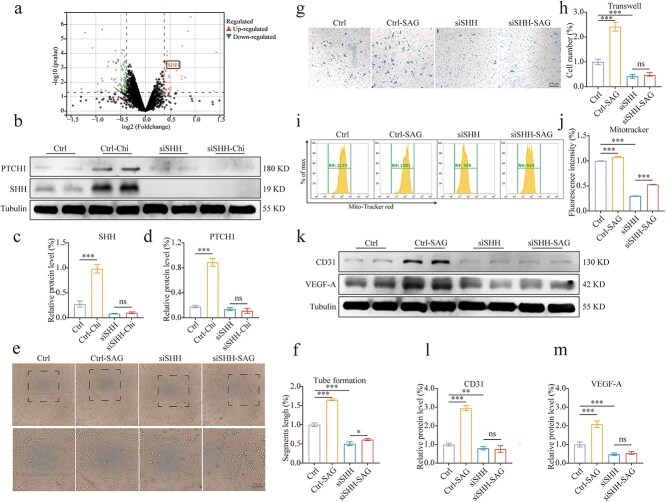
PPAR signaling promotes HUVEC function via SHH expression. (**a**) Volcano map of DEGs (adj. *p* < 0.01 based on sequencing results). (**b, c, d**) Cells were transfected with SHH small interfering RNA (siSHH) for 24 h, treated with 200 μg/ml AGEs for 24 h, and then exposed to 500 μmol SAG for 24 h. Western blot was used to detect the relative levels of SHH and PTCH1 protein in HUVECs after Chi intervention in the presence or absence of siSHH, *n* = 4. (**e, f**) Tube formation assays were used to assess segment length and HUVECs’ tube formation ability, *n* = 3 (scale bar: 100 μm). (**g, h**) Transwell assays were conducted for evaluation of HUVEC migration, *n* = 3 (scale bar: 100 μm). (**i, j**) MitoTracker™ Red CMXRos was measured by flow cytometry after siSHH and SAG intervention, *n* = 3. (**k, l, m**) The relative levels of CD31 and VEGF-A in HUVECs after siSHH and SAG intervention were measured by western blot, *n* = 4. The results are expressed as mean ± SD. ^*^p < 0.05, ^**^p< 0.01, ^***^p < 0.001; ns, not significant. *PPAR* peroxisome proliferator-activated receptor, *HUVECs* human umbilical vein endothelial cells, *OXPHOS* oxidative phosphorylation, *AGEs* advanced glycation end-products, *SD* standard deviation, *VEGF-A* vascular endothelial growth factor A, *SHH* sonic hedgehog signaling

Given the relationship between PPAR activation and mitochondrial function, we further investigated the pathways through which PPARs target mitochondria. Upon re-analysis of the sequencing data, we prioritized the SHH signaling pathway (Figure 6a). Recent studies have highlighted the important role of SHH in mitochondrial energy metabolism [25]. We verified that the activation of PPARs under pathological conditions can initiate the SHH signaling pathway. This finding was corroborated through experiments conducted on HUVECs (Figure S4a, b, see online supplementary material) and in the epidermal tissue at the periphery of wounds (Figure S4c–e, see online supplementary material). Additionally, FISH experiments revealed that SHH predominantly originates from vascular endothelial cells (Figure S3g–i, see online supplementary material). However, this activation effect was attenuated upon the knockdown of SHH using siRNA (Figure 6b–d). Validation using SAG, a specific SHH activator, indicated that SHH activation effectively promoted tube formation and cell migration, effects that were abolished following SHH knockdown (Figure 6e–h). We also assessed the impact of SHH activation on mitochondrial function; MitoTracker™ Red CMXRos assay demonstrated that SHH activation enhanced mitochondrial activity, but this effect was inhibited by SHH knockdown (Figure 6i, j). Similarly, mPTP assay and JC-10 assay yielded consistent results (Figure S5a–d, see online supplementary material). Lastly, using SAG, we demonstrated that SHH activation can promote the expression of CD31 and VEGF-A in HUVECs (Figure 6k–m). Taken together, these results have suggested that PPAR activation has enhanced HUVEC function, potentially via the SHH signaling pathway.

### SHH mediates PPAR signaling to promote OXPHOS and regulate HUVEC function

**Figure 7:**
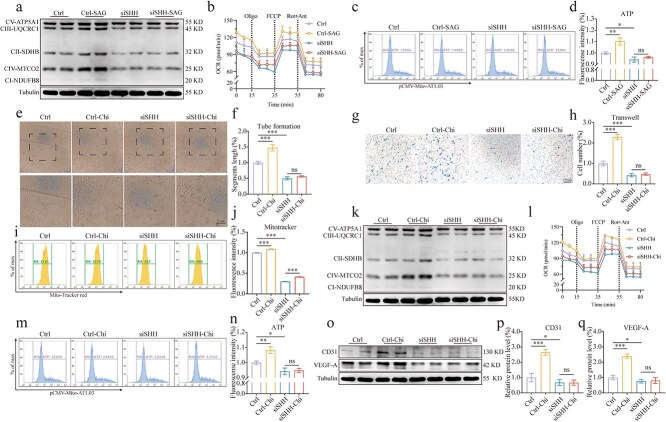
SHH mediates PPAR signaling to promote OXPHOS and regulate HUVEC function. (**a**) Cells were transfected with siSHH for 24 h, treated with 200 μg/ml AGEs for 24 h, and then exposed to 500 μM SAG for 24 h. The relative levels of mitochondrial OXPHOS in HUVECs, *n* = 3. (**b**) OCR and maximal respiration response of HUVECs were measured with Seahorse after SAG intervention, *n* = 3. (**c, d**) Flow cytometry was performed using mitochondrial ATP fluorescent probes after SAG intervention, *n* = 3. (**e, f**) Tube formation assays were used to assess segment length and HUVECs’ tube formation ability after Chi intervention, *n* = 3 (scale bar: 100 μm). (**g, h**) Transwell assays were conducted for evaluation of HUVEC migration after Chi intervention, *n* = 3 (scale bar: 100 μm). (**i, j**) MitoTracker™ Red CMXRos was measured by flow cytometry after Chi intervention, *n* = 3. (**k**) The relative levels of mitochondrial OXPHOS in HUVECs after Chi intervention were measured by western blot, *n* = 3. (**l**) OCR and maximal respiration response of HUVECs were measured with Seahorse after Chi intervention, *n* = 3. (**m, n**) Flow cytometry was performed using mitochondrial ATP fluorescent probes after Chi intervention, *n* = 3. (**o, p, q**) The relative levels of CD31 and VEGF-A in HUVECs after Chi intervention were measured by western blot, *n* = 4. The results are expressed as mean ± SD. ^*^p < 0.05, ^**^p < 0.01, ^***^p < 0.001; ns, not significant. *PPAR* peroxisome proliferator-activated receptor, *HUVECs* human umbilical vein endothelial cells, *OXPHOS* oxidative phosphorylation, *AGEs* advanced glycation end-products, *SD* standard deviation, *VEGF-A* vascular endothelial growth factor A, *SHH* sonic hedgehog signaling, *OCR* oxygen consumption rate

**Figure 8:**
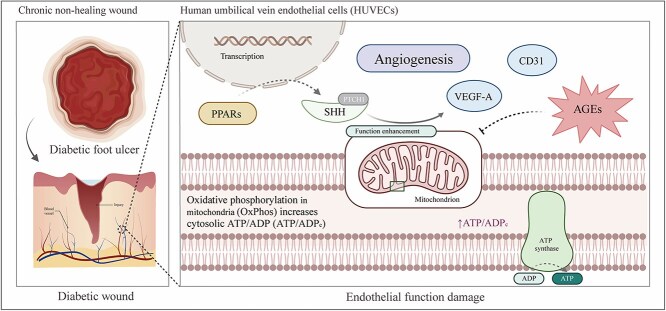
Schematic of the molecular mechanism (created with BioRender.com)

To further verify our hypothesis, we reactivated the SHH signaling following SHH knockdown and assessed its effect on mitochondrial OXPHOS via western blot. The results demonstrated that OXPHOS increased following SHH signaling activation, but this effect was significantly inhibited by SHH knockdown (Figure 7a). To investigate the role of SHH signaling in the OXPHOS process, the Seahorse extracellular flux analyzer was used to analyze the cellular oxygen consumption rate (OCR). The results showed that SHH stimulation increased the basal OCR and the maximal respiratory response of HUVECs (Figure 7b). In addition, flow cytometry with a mitochondrial ATP fluorescent probe further confirmed the regulatory role of SHH in mitochondrial energy metabolism (Figure 7c, d). Based on these results, we hypothesize that SHH signaling mediates PPAR activation, thereby enhancing mitochondrial OXPHOS and promoting cell function. We activated PPARs following SHH knockdown, and the results showed that the tube formation effect induced by PPAR activation was inhibited following SHH knockdown (Figure 7e, f). The Transwell assay was consistent with those of the tube formation assay (Figure 7g, h). Additionally, MitoTracker™ Red CMXRos assay indicated that the enhancement of mitochondrial activity by PPAR activation was inhibited by SHH knockdown (Figure 7i, j). This result was corroborated by mPTP assay and JC-10 assay (Figure S5e–h, see online supplementary material). Western blot analysis revealed that the PPAR activation–induced enhancement of mitochondrial OXPHOS was significantly attenuated upon SHH knockdown (Figure 7k). Consistent with this finding, PPAR activation markedly elevated both the basal OCR and the maximal respiratory capacity in HUVECs (Figure 7l). This trend was further corroborated by flow cytometric analysis using a mitochondrial ATP-specific fluorescent probe (Figure 7m, n). Furthermore, SHH knockdown effectively suppressed the upregulation of CD31 and VEGF-A, which were otherwise promoted by PPAR activation (Figure 7o–q). Overall, our findings have suggested that the SHH signaling pathway has mediated PPAR activation and enhanced endothelial cell function by promoting mitochondrial OXPHOS and overall mitochondrial function. We also developed a model outlining the mechanism by which PPAR activation promotes the healing process of diabetic wounds (Figure 8).

## Discussion

The mechanisms underlying the difficulty in healing DFU and the onset and progression of diabetes remain unclear. It is still unknown whether drugs that treat diabetes through different mechanisms have an impact on wound healing. Elucidating their additional benefits holds significant clinical importance [1, 26]. Thus, identifying and characterizing drugs that expedite diabetic wound healing is essential for enhancing the quality of life for patients with DFU. In this work, we have demonstrated that activation of PPARs accelerates diabetic wound healing by promoting angiogenesis and regulating endothelial cell function. Mechanistic investigations revealed that the SHH signaling pathway mediates PPAR activation, enhancing endothelial cell function by promoting mitochondrial OXPHOS and overall mitochondrial function. These findings elucidate the mechanism of action for PPAR-related agonists in diabetic wound treatment and suggest that PPAR activation could offer additional therapeutic benefits for DFU, signifying its potential clinical application.

Wound healing is a multifaceted biological process that involves angiogenesis, collagen production, cell migration, and proliferation [19, 27]. Effective wound healing necessitates a coordinated effort among angiogenic cytokines, endothelial cells, and extracellular matrix components [28]. In the pathological context of DFUs, endothelial dysfunction substantially impairs the normal wound healing process [29]. Impaired angiogenesis is often associated with disrupted signaling pathways and aberrant gene expression induced by diabetes [30]. PPARs are classified into three primary subtypes: PPARα, PPARβ/δ, and PPARγ. The expression of PPARs in endothelial cells was first reported two decades ago [31], and all PPAR subtypes have been implicated in the modulation of angiogenesis [32]. Our research demonstrates that the expression of the PPAR family is significantly diminished in the neovascularization of skin wounds in patients with DFU, suggesting that PPAR signaling may play a critical role in diabetic wound healing. PPAR activators, such as chiglitazar, enhance PPAR protein expression primarily through transcriptional activation and post-translational modification of PPARs. Chiglitazar exhibits transactivating activity across PPARα, PPARγ, and PPARδ subtypes [8, 33, 34]. Additionally, chiglitazar inhibits the ubiquitination of PPARs, thereby increasing protein stability [35]. This feedback mechanism provides a plausible explanation for the substantial upregulation of PPAR protein levels observed in endothelial cells and wound tissues following chiglitazar treatment, effectively mitigating the pathological downregulation observed in the DFU microenvironment.

PPARs control gene expression linked to energy metabolism, cell development, and differentiation, making their biological role crucial in medical research and drug discovery [36, 37]. Clinically, PPAR agonists have significant relevance across metabolic diseases, chronic inflammatory diseases, autoimmune disorders, neurological and psychiatric conditions, and malignancies [10, 11, 38]. From a metabolic standpoint, the activation of PPARs is widely employed in the treatment of diabetes and dyslipidemia, owing to its efficacy in enhancing insulin sensitivity and modulating lipid distribution [39]. Beyond metabolic contexts, numerous studies have demonstrated that the activation of various PPAR subtypes can mitigate inflammatory responses, consequently enhancing glycolipid metabolism and mitochondrial energy metabolism [40–43]. More importantly, clinical data indicate that the activation of PPARs not only ameliorates hyperglycemia and obesity but also confers vascular protection. This dual action offers therapeutic advantages for managing cardiovascular and macrovascular complications associated with diabetes, thereby demonstrating an additional benefit in the treatment regimen [44–47]. The role of individual PPAR subtypes in angiogenesis presents a fascinating paradox between *in vitro* and *in vivo* findings, a nuance critical to interpreting our results. *In vitro* studies using endothelial cells typically indicate that under physiological conditions, PPARα and PPARγ activation can exhibit anti-angiogenic properties, while PPARβ/δ is more consistently pro-angiogenic [31, 32]. However, this narrative is compellingly reversed in the context of complex disease states like diabetes and ischemia. A substantial body of *in vivo* evidence demonstrates that activation of all three PPAR subtypes—via agonists like fenofibrate (PPARα) [48–51], rosiglitazone (PPARγ) [52–55], and GW501516 (PPARβ/δ) [56, 57]—confers potent protection against endothelial dysfunction and robustly promotes reparative angiogenesis. The observed discrepancies can be attributed to the fact that angiogenesis within a pathological microenvironment is modulated not only by endothelial cells but also through interactions with immune and stromal cells, as well as systemic metabolic factors. Consequently, apparently inconsistent results underscore the critical importance of biological context. Our findings demonstrate that the pan-PPAR agonist chiglitazar significantly enhances angiogenesis and wound healing, thereby validating this *in vivo* model. By activating all three PPAR subtypes, a pan-agonist can elicit a coordinated response that overcomes the limitations associated with targeting a single subtype, thereby facilitating effective healing in diabetic wounds.

In this work, we observed a downward trend in fasting blood glucose levels in mice treated with PPAR activator, although the change was not statistically significant. This suggests that the benefits of PPAR activation on wound healing may not be attributable to its hypoglycemic effects [43, 58]. The lack of significant change in blood glucose levels could be due to several factors, including the diabetes model used, treatment duration and dosage, wound formation, and systemic or local drug absorption, all of which warrant further investigation.

The primary aim of this work was to demonstrate how PPAR activators influence endothelial cells at the wound site to promote wound healing. Activation of PPARs initiates transcriptional processes that produce downstream effects in endothelial cells, aligning with the role of the PPAR family as transcription factors interacting with target gene promoters [11]. We identified the SHH signaling pathway as a key mediator that enhances endothelial cell proliferation, migration, and adhesion following PPAR activation. The SHH signaling pathway, a well-conserved pathway, is crucial in embryonic development, stem cell maintenance, and tissue homeostasis [59, 60]. Studies have demonstrated that the activated SHH signaling pathway plays a crucial role in regulating cell proliferation, migration, and adhesion [61–64]. In contrast, our findings reveal that SHH signaling in endothelial cells is markedly suppressed in diabetic conditions, aligning with previous research [65]. FISH analysis showed a significant co-localization of SHH mRNA with CD31^+^ endothelial cells within the wound neovasculature, indicating the presence of an endothelial-autonomous PPARs–SHH signaling axis. Nonetheless, we recognize the complexity of the wound microenvironment, where other stromal cells, such as fibroblasts [63, 66] and macrophages [64, 67], also produce SHH and contribute to tissue repair through paracrine mechanisms. Consequently, while our data robustly demonstrate that PPAR activation directly enhances SHH expression in endothelial cells, we cannot rule out the possibility that additional paracrine signals from other cellular sources may further potentiate the SHH-mediated pro-healing effects observed. Further analysis of the energy system in endothelial cells revealed that SHH significantly increases mitochondrial OXPHOS, providing the necessary energy for cell proliferation and angiogenesis. This finding is consistent with previous reports indicating that OXPHOS supports and enhances angiogenic activity [68]. Additionally, SHH has been shown to promote OXPHOS in neurons and protect them from various stressors [69]. Further studies have demonstrated that SHH signaling reduces mitochondrial fission and promotes mitochondrial fusion and elongation, thereby enhancing overall mitochondrial function [70]. Overall, mitochondrial dynamics, including fission and fusion, are crucial for maintaining mitochondrial phenotype and function [71]. For instance, adverse morphological changes in mitochondria within endothelial cells can trigger glycolysis and re-establishment of the mitochondrial membrane potential [72]. Moreover, reducing mitochondrial fission has been shown to improve mitochondrial function and promote endothelial activity [73].

While our study has established that the PPARs/SHH signaling axis plays a role in regulating wound healing in diabetes, certain limitations remain. First, the utilization of drug-induced pan-activation of PPARs may obscure the distinct contributions of individual receptor subtypes, notwithstanding our animal studies that indicate enhanced wound healing. Second, while we have demonstrated that PPAR activation leads to upregulation of SHH expression, the exact molecular mechanism—whether through direct transcriptional regulation of the SHH gene via a PPRE or through an indirect pathway involving other intermediates—remains to be fully elucidated. Identifying the direct transcriptional targets of PPARs that facilitate this cross-talk is a critical next step. Lastly, translating these promising preclinical findings into clinical practice necessitates large-scale, rigorously controlled trials to specifically assess the efficacy and safety of PPAR agonists for DFU treatment. Future research should prioritize validating the clinical efficacy of PPAR-related therapies for DFU treatment, including the dissection of individual subtype contributions and the detailed molecular connection to SHH signaling.

In sum, the complex process of diabetic wound repair is highly challenging, and there is a growing eagerness to explore new methods to promote healing [4, 74]. Our findings provide novel insights into the mechanism by which PPAR signaling activation promotes endothelial cell function and diabetic wound healing, primarily through the regulation of mitochondrial OXPHOS via SHH signaling. These insights provide a solid evidence base for the future clinical application of PPAR-related agonists in DFU therapy.

## Conclusions

Our study demonstrates that activation of PPARs significantly enhances diabetic wound healing by promoting angiogenesis and improving endothelial cell function. The underlying mechanism involves the SHH signaling pathway, which mediates the effects of PPAR activation by enhancing mitochondrial OXPHOS and overall mitochondrial function. These findings offer valuable insights into the therapeutic potential of PPAR agonists in managing DFU. Future research should focus on validating the clinical efficacy of PPAR-related therapies for DFU treatment.

## Abbreviations

cDNA, complementary DNA; AGEs, advanced glycation end-products; Chi, chiglitazar; DFU, diabetic foot ulcer; CD31, platelet endothelial cell adhesion molecule-1; GEO, Gene Expression Omnibus; STZ, streptozotocin; HUVECs, human umbilical vein endothelial cells; mPTP, mitochondrial permeability transition pore; VEGF-A, vascular endothelial growth factor A; OXPHOS, oxidative phosphorylation; PPARs, peroxisome proliferator-activated receptors; qRT-PCR, quantitative reverse transcription polymerase chain reaction; Ros, rosiglitazone; T2DM, type 2 diabetes mellitus; SHH, sonic hedgehog signaling; OCR, oxygen consumption rate; FISH, fluorescence *in situ* hybridization.

## Supplementary Material

Supplementary_Fig-1_tkaf063

Supplementary_Fig-2_tkaf063

Supplementary_Fig-3_tkaf063

Supplementary_Fig-4_tkaf063

Supplementary_Fig-5_tkaf063

Supplementary_Table_1_tkaf063

Supplementary_Fig_Legend_tkaf063

## Data Availability

The datasets used and/or analyzed during this study are available from the corresponding author on reasonable request.
