# Supplementary material for: Peroxisome proliferator-activated receptors-mediated diabetic wound healing regulates endothelial cells’ mitochondrial function via sonic hedgehog signaling
Source: Burns Trauma. 2025 Sep 10;13:tkaf063. doi: 10.1093/burnst/tkaf063 (PMC12597028; doi:10.1093/burnst/tkaf063)
Supplement: Supplementary_Fig-1_tkaf063 [file supplementary_fig-1_tkaf063.pdf]

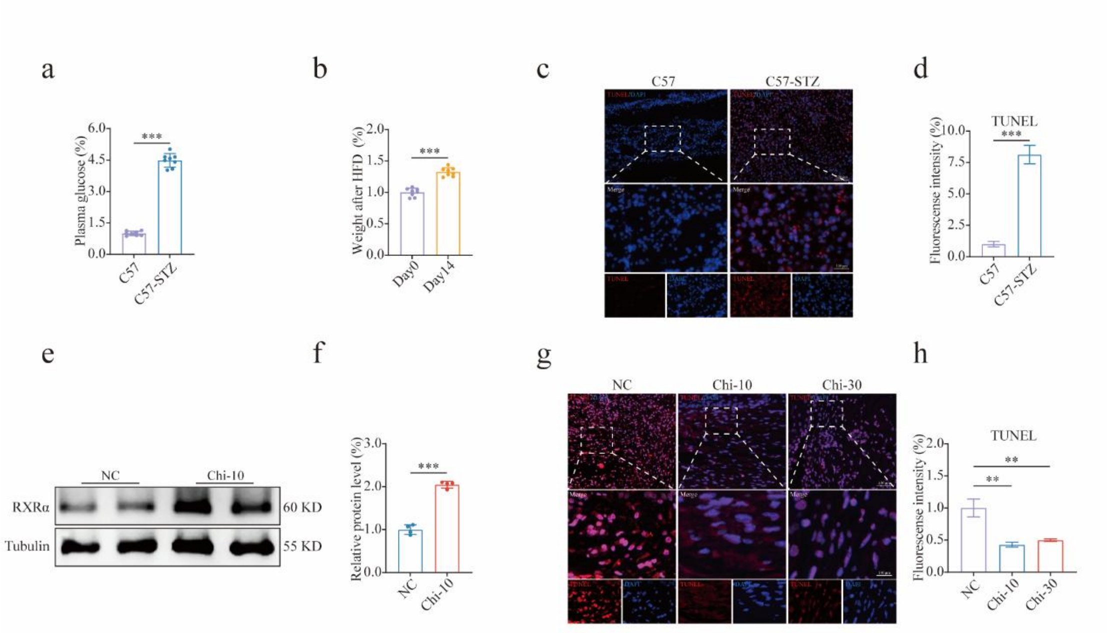

Supplementary Fig. S1. Effect of Chi intervention on glucose levels, body weight, and cellular apoptosis in diabetic mice. **(a)** One week after STZ injection, plasma glucose was measured, n = 8. **(b)** Weight change after 30 days of HDF diet, n = 8. **(c, d)** Representative immunofluorescence images and analyses showed pancreatic apoptosis after intraperitoneal injection of STZ, n = 4 (scale bar: 500 μm). **(e, f)** The relative levels of RXRα in diabetic wounds on day 9 within the NC group and Chi-10 group, n = 4. **(g, h)** Representative immunofluorescence images and analyses showing cellular apoptosis in skin wounds on day 9 after Chi intervention. n = 3 (scale bar: 400 μm). The results were expressed as mean ± SD. * $p < 0.05$, ** $p < 0.01$, *** $p < 0.001$; ns, not significant.
