# Supplementary material for: Peroxisome proliferator-activated receptors-mediated diabetic wound healing regulates endothelial cells’ mitochondrial function via sonic hedgehog signaling
Source: Burns Trauma. 2025 Sep 10;13:tkaf063. doi: 10.1093/burnst/tkaf063 (PMC12597028; doi:10.1093/burnst/tkaf063)
Supplement: Supplementary_Fig-2_tkaf063 [file supplementary_fig-2_tkaf063.pdf]

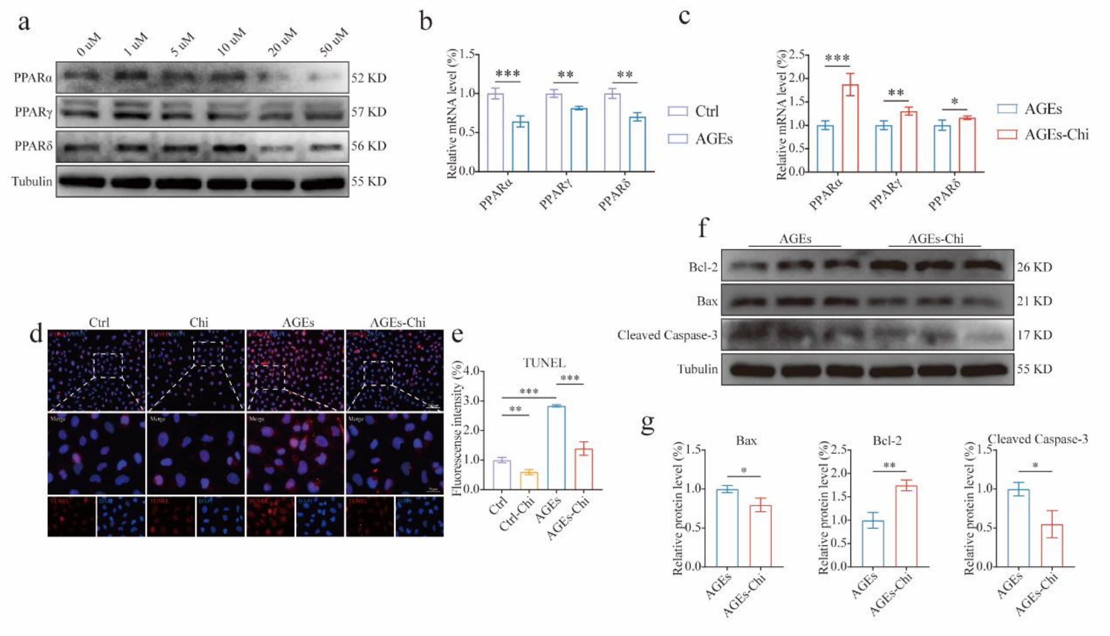

Supplementary Fig. S2. Chi intervention regulates PPARs expression and apoptosis in HUVECs under normal and pathological conditions. (a) Western blot determined the optimal PPARs activation concentration post Chi intervention. **(b)** A qRT-PCR was used to analyze the relative expression of PPARs mRNA in HUVECs after Chi intervention under normal conditions, n = 4. **(c)** A qRT-PCR was used to analyze the relative expression of PPARs mRNA in HUVECs after Chi intervention under pathological conditions, n = 4. **(d, e)** TUNEL staining and statistical analyses in HUVECs, cells were pretreated with 200 μg/ml AGEs for 24 hours, then exposed to 1 μM Chi for 48 hours, n = 4 (scale bar: 200 μm). **(f, g)** The relative levels of Bax, Bcl-2 and Cleaved Caspase-3 in HUVECs after Chi intervention under pathological conditions were measured by Western blot, n = 3. The results were expressed as mean ± SD. * $p < 0.05$, ** $p < 0.01$, *** $p < 0.001$; ns, not significant.
