# Supplementary material for: Peroxisome proliferator-activated receptors-mediated diabetic wound healing regulates endothelial cells’ mitochondrial function via sonic hedgehog signaling
Source: Burns Trauma. 2025 Sep 10;13:tkaf063. doi: 10.1093/burnst/tkaf063 (PMC12597028; doi:10.1093/burnst/tkaf063)
Supplement: Supplementary_Fig-3_tkaf063 [file supplementary_fig-3_tkaf063.pdf]

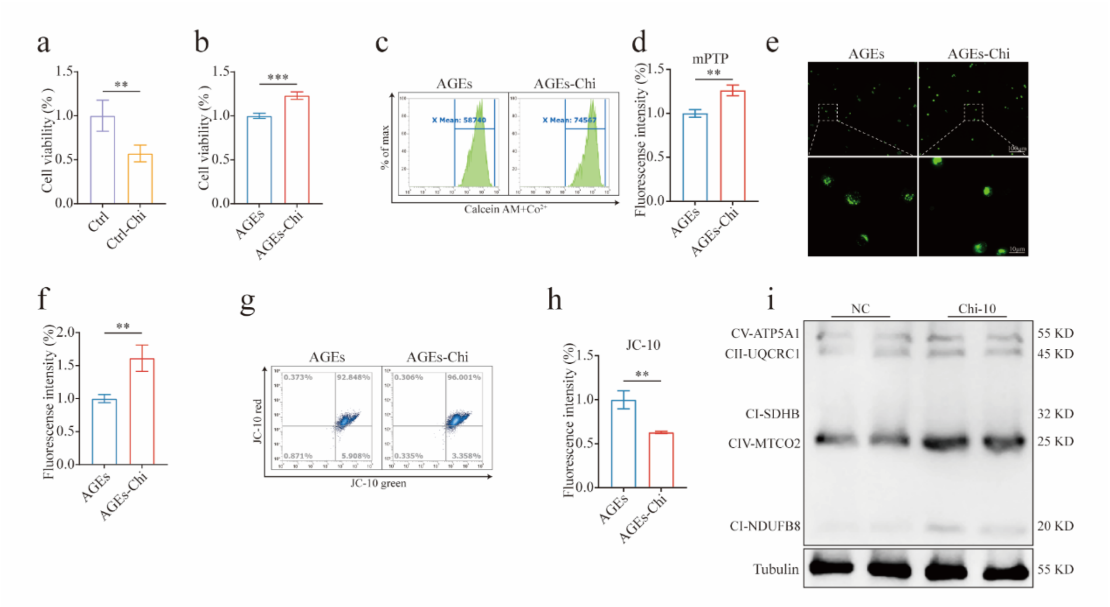

Supplementary Fig. S3. Effects of Chi intervention on cell viability and mitochondrial function in HUVECs under normal and pathological conditions. **(a)** Cell viability in HUVECs after 48 hours of Chi intervention under normal conditions was assessed using the CCK-8 assay, n = 4. **(b)** Cell viability in HUVECs after 48 hours of Chi intervention under pathological conditions was assessed using the CCK-8 assay, n = 4. **(c, d)** Flow cytometry measured mPTP analysis under pathological conditions, n = 3. **(e, f)** Representative immunofluorescence images and analyses for mPTP (Green) in HUVECs under pathological conditions, n = 3 (scale bar: 100 μm). **(g, h)** Flow cytometry measured JC-10 analysis under pathological conditions, n = 3. **(i)** The relative levels of mitochondrial OXPHOS in diabetic wounds on day 9 within the NC group and Chi-10 group. The results were expressed as mean ± SD. * $p < 0.05$, ** $p < 0.01$, *** $p < 0.001$; ns, not significant.
