# Supplementary material for: Peroxisome proliferator-activated receptors-mediated diabetic wound healing regulates endothelial cells’ mitochondrial function via sonic hedgehog signaling
Source: Burns Trauma. 2025 Sep 10;13:tkaf063. doi: 10.1093/burnst/tkaf063 (PMC12597028; doi:10.1093/burnst/tkaf063)
Supplement: Supplementary_Fig-4_tkaf063 [file supplementary_fig-4_tkaf063.pdf]

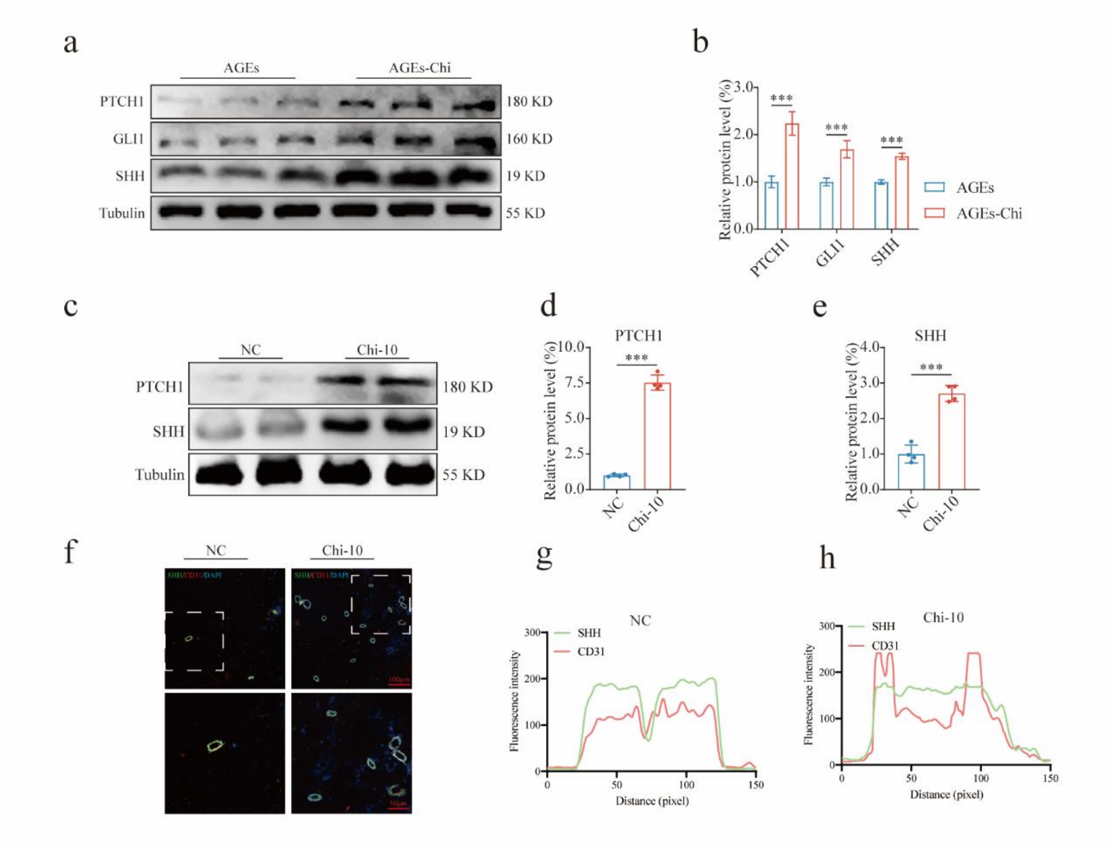

Supplementary Fig. S4. Chi intervention modulates SHH signaling in HUVECs and diabetic wounds. (a, b) The relative levels of PTCH1, GLI1 and SHH in HUVECs after Chi intervention under pathological conditions were measured by Western blot, n = 3. (**c, d, e**) Western blot analysis was performed to assess the levels of SHH and PTCH1 in diabetic wounds on day 9 within the NC group and Chi-10 group, n = 4. (**f**) the expression of SHH and CD31 mRNA levels measured by FISH in diabetic wounds on day 9 by confocal fluorescence microscopy. (**g, h**) Plots of normalized fluorescence intensity profiles collected by confocal microscopy and processed in ImageJ (n = 4; Scale bar, 50 μm). The results were expressed as mean ± SD. * $p < 0.05$, ** $p < 0.01$, *** $p < 0.001$; ns, not significant.
