# Supplementary material for: Peroxisome proliferator-activated receptors-mediated diabetic wound healing regulates endothelial cells’ mitochondrial function via sonic hedgehog signaling
Source: Burns Trauma. 2025 Sep 10;13:tkaf063. doi: 10.1093/burnst/tkaf063 (PMC12597028; doi:10.1093/burnst/tkaf063)
Supplement: Supplementary_Fig-5_tkaf063 [file supplementary_fig-5_tkaf063.pdf]

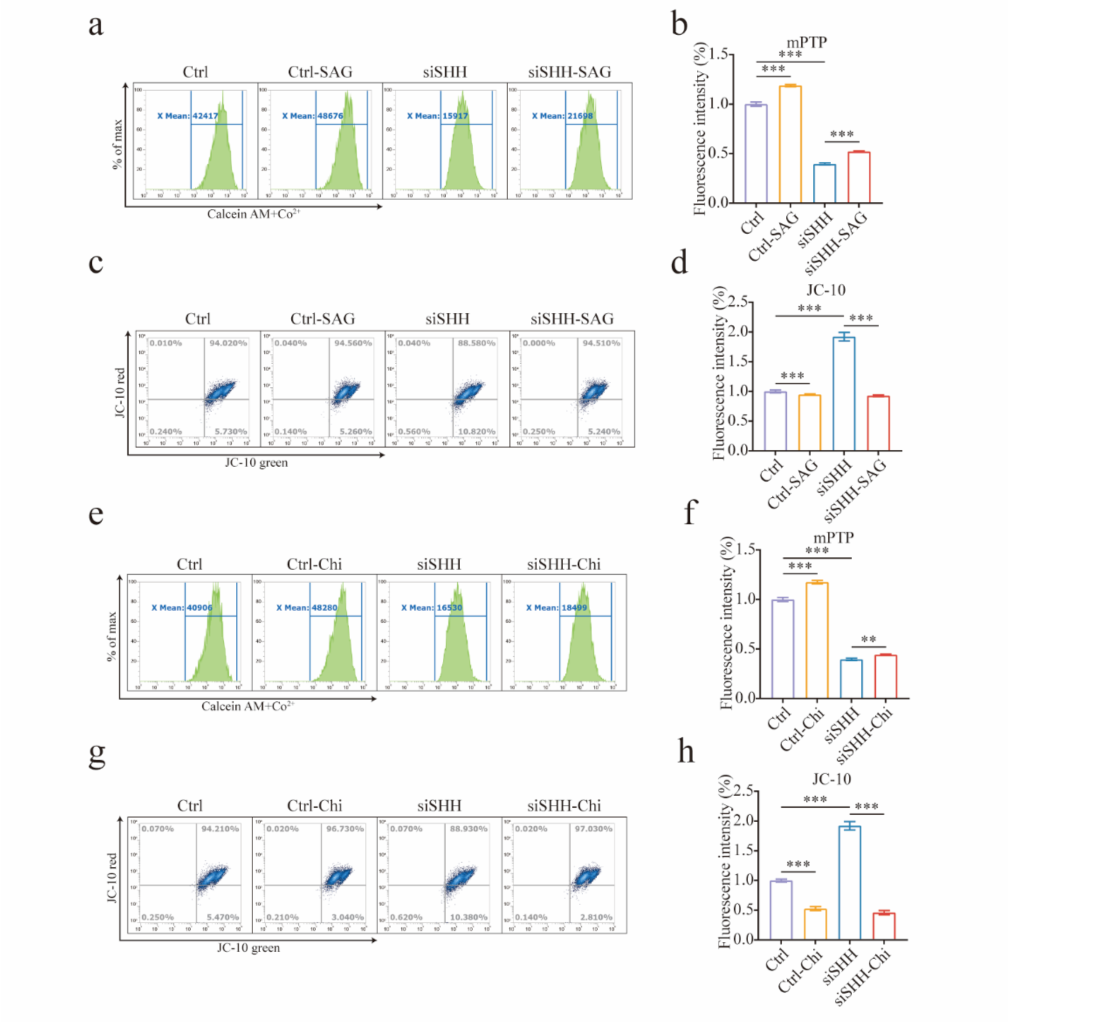

Supplementary Fig. S5. Effects of SHH silencing, SAG activation, and Chi intervention on mitochondrial function in HUVECs. (a, b, c, d) Flow cytometry measured mPTP and JC-10. Cells were transfected with siSHH for 24 hours, treated with 200 µg/mL AGEs for 24 hours, and then exposed to 500 µM SAG for 24 hours, n = 3. **(e, f, g, h)** Flow cytometry measured mPTP and JC-10. Cells were pretreated with 200 µg/ml AGEs for 24 hours, then exposed to 1 µM Chi for 48 hours, n = 3. The results were expressed as mean ± SD. * $p < 0.05$, ** $p < 0.01$, *** $p < 0.001$; ns, not significant.
