# Supplementary material for: Peroxisome proliferator-activated receptors-mediated diabetic wound healing regulates endothelial cells’ mitochondrial function via sonic hedgehog signaling
Source: Burns Trauma. 2025 Sep 10;13:tkaf063. doi: 10.1093/burnst/tkaf063 (PMC12597028; doi:10.1093/burnst/tkaf063)
Supplement: Supplementary_Table_1_tkaf063 [file supplementary_table_1_tkaf063.pdf]

Supplementary Table 1: RT-qPCR primers were used in this study

Name	Forward sequence	Reverse sequence
Human- PPARα	ATGGTGGACACGGAAAGCC	CGATGGATTGCGAAATCTCTTGG
Human- PPARγ	TACTGTCGGTTTCAGAAATGCC	GTCAGCGGACTCTGGATTCAG
Human- PPARβ/δ	GCCTCTATCGTCAACAAGGAC	GCAATGAATAGGGCCAGGTC
Human-β-Actin	CATGTACGTTGCTATCCAGGC	CTCCTTAATGTCACGCACGAT
