# Supplementary material for: Peroxisome proliferator-activated receptors-mediated diabetic wound healing regulates endothelial cells’ mitochondrial function via sonic hedgehog signaling
Source: Burns Trauma. 2025 Sep 10;13:tkaf063. doi: 10.1093/burnst/tkaf063 (PMC12597028; doi:10.1093/burnst/tkaf063)
Supplement: Supplementary_Fig_Legend_tkaf063 [file supplementary_fig_legend_tkaf063.docx]

**Supplementary Fig. S1.** **Effect of Chi intervention on pancreatic apoptosis, cellular apoptosis in skin wounds, and PPARs activation in HUVECs. (a)** One week after STZ injection, plasma glucose was measured, n = 8. **(b)** Weight change after 30 days of HDF diet, n = 8. **(c, d)** Representative immunofluorescence images and analyses showed pancreatic apoptosis after intraperitoneal injection of STZ, n = 4 (scale bar: 500 μm). **(e, f)** Representative immunofluorescence images and analyses showing cellular apoptosis in skin wounds on day 9 after Chi intervention. n = 3 (scale bar: 400 μm). **(g)** Western blot determined the optimal PPARs activation concentration post Chi intervention. **(h)** A qRT-PCR was used to analyze the relative expression of PPARs mRNA in HUVECs after Chi intervention under normal conditions, n = 4. **(i)** A qRT-PCR was used to analyze the relative expression of PPARs mRNA in HUVECs after Chi intervention under pathological conditions, n = 4. **(j, k)** TUNEL staining and statistical analyses in HUVECs, cells were pretreated with 200 μg/ml AGEs for 24 hours, then exposed to 1 μM Chi for 48 hours, n = 4 (scale bar: 200 μm). **(l, m, n, o)** The relative levels of Bax, Bcl-2 and Cleaved Caspase-3 in HUVECs after Chi intervention under pathological conditions were measured by Western blot, n = 3. The results were expressed as mean ± SD. * *p* < 0.05, ** *p* <0.01, *** *p* < 0.001; ns, not significant.

**Supplementary Fig. S2. Analysis of mitochondrial phenotype in HUVECs under Chi intervention. (a)** Cell viability in HUVECs after 48 hours of Chi intervention under normal conditions was assessed using the CCK-8 assay, n = 4. **(b)** Cell viability in HUVECs after 48 hours of Chi intervention under pathological conditions was assessed using the CCK-8 assay, n = 4. **(c, d)** Flow cytometry measured mPTP analysis under pathological conditions, n = 3. **(e, f)** Representative immunofluorescence images and analyses for mPTP (Green) in HUVECs under pathological conditions, n = 3 (scale bar: 100 μm). **(g, h)** Flow cytometry measured JC-10 analysis under pathological conditions, n = 3. **(i, j)** The relative levels of PTCH1, GLI1 and SHH in HUVECs after Chi intervention under pathological conditions were measured by Western blot, n = 3. **(k, l, m, n)** Flow cytometry measured mPTP and JC-10. Cells were transfected with siSHH for 24 hours, treated with 200 µg/mL AGEs for 24 hours, and then exposed to 500 µM SAG for 24 hours, n = 3. **(o, p, q, r)** Flow cytometry measured mPTP and JC-10. Cells were pretreated with 200 μg/ml AGEs for 24 hours, then exposed to 1 μM Chi for 48 hours, n = 3. The results were expressed as mean ± SD. * *p* < 0.05, ** *p* <0.01, *** *p* < 0.001; ns, not significant.

**Supplementary Fig. S3. Assessment of mitochondrial OXPHOS, RXRα, SHH, and PTCH1 expression in diabetic wounds after Chi intervention. (a)** The relative levels of mitochondrial OXPHOS in diabetic wounds on day 9 within the NC group and Chi-10 group. **(b, c)** The relative levels of RXRα in diabetic wounds on day 9 within the NC group and Chi-10 group, n = 4. **(d, e, f)** Western blot analysis was performed to assess the levels of SHH and PTCH1 in diabetic wounds on day 9 within the NC group and Chi-10 group, n = 4. **(g)** the expression of SHH and CD31 mRNA levels measured by FISH in the in diabetic wounds on day 9 by confocal fluorescence microscopy. **(h i)** Plots of normalized fluorescence intensity profiles collected by confocal microscopy and processed in ImageJ (n = 4; Scale bar, 50 μm). The results were expressed as mean ± SD. * *p* < 0.05, ** *p* <0.01, *** *p* < 0.001; ns, not significant.
